# Combining two-directional synthesis and tandem reactions, part 11: second generation syntheses of (±)-hippodamine and (±)-epi-hippodamine

**DOI:** 10.1186/1860-5397-4-4

**Published:** 2008-01-17

**Authors:** Annabella F Newton, Martin Rejzek, Marie-Lyne Alcaraz, Robert A Stockman

**Affiliations:** 1 School of Chemistry, University of Nottingham, Nottingham NG7 2RD, UK; 2 School of Chemical Sciences and Pharmacy, University of East Anglia, Norwich NR4 7TJ, UK; 3 AstraZeneca, Bakewell Road, Loughborough, Leics LE11 5RH, UK

## Abstract

**Background:**

Hippodamine is a volatile defence alkaloid isolated from ladybird beetles which holds potential as an agrochemical agent and was the subject of a synthesis by our group in 2005.

**Results:**

Two enhancements to our previous syntheses of (±)-hippodamine and (±)-epi-hippodamine are presented which are able to shorten the syntheses by up to two steps.

**Conclusions:**

Key advances include a two-directional homologation by cross metathesis and a new tandem reductive amination / double intramolecular Michael addition which generates 6 new bonds, 2 stereogenic centres and two rings, giving a single diastereomer in 74% yield.

## Background

**Figure 1:**
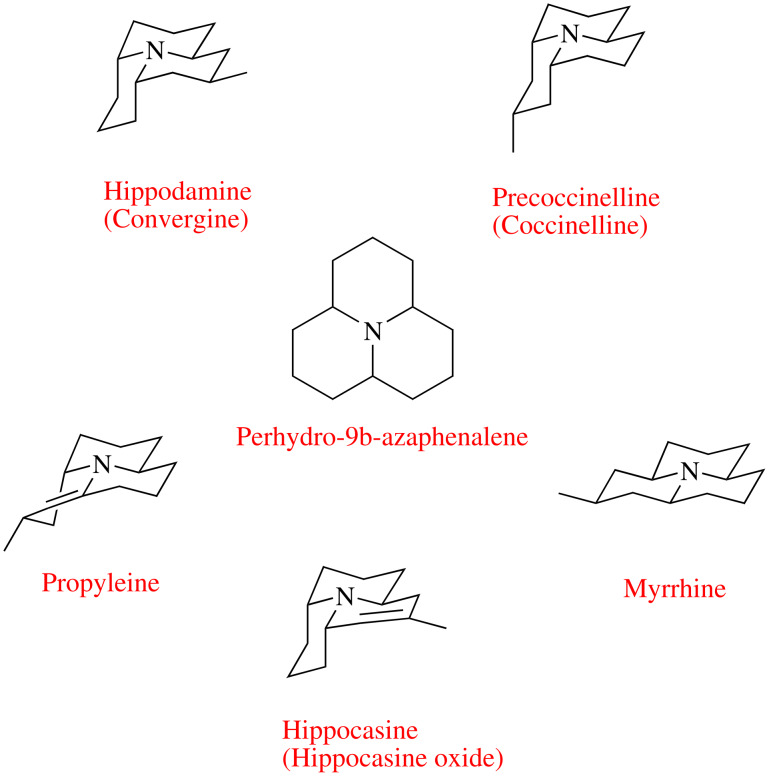
Structures of Coccinellid Alkaloids (*N*-Oxides and their names in brackets).

Ladybird beetles (Coleoptera: Coccinellidae) are important predators contributing to the natural control of pest aphid populations and are therefore of considerable commercial interest. However, ladybirds themselves are attacked by a range of natural enemies. General predation on ladybirds by vertebrates such as birds is largely prevented by highly toxic defence alkaloids contained in a reflex bleed released when the ladybird is attacked. To date, eight alkaloids of this type have been isolated from coccinellid beetles [1], all of them being formally derivatives of perhydro-9b-azaphenalene (Figure 1). Another group of natural enemies, parasitic insects, can cause substantial reductions in populations of ladybird species. Recent research [2] has shown that the parasites locate the ladybirds through perception of certain defence alkaloids that they emit. If ladybirds are to be used effectively in insect pest control then their parasites must be controlled as well. The significant attraction of parasitic insects to the ladybird alkaloids suggests that there is potential for development of control strategies for this particular natural enemy. To further test this theory significant amounts of the defensive alkaloids will be needed. Coccinelid beetles seem to be the sole source of the defence alkaloids. Consequently much attention has been paid to developing syntheses of these compounds.

**Scheme 1:**
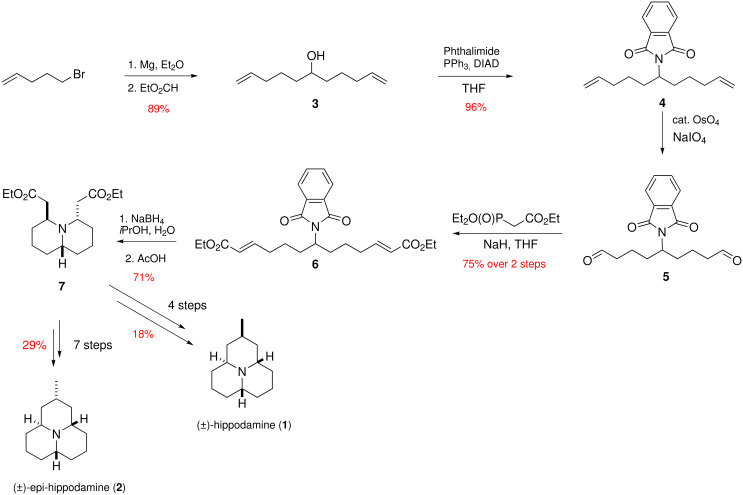
Summary of our previous syntheses of hippodamine (**1**) and epi-hippodamine (**2**).

Hippodamine (**1**) is a naturally occurring alkaloid isolated from a ladybird beetle *Hippodamia convergens* by Tursch and co-workers in 1972 [3]. The structure of hippodamine (**1**) was established two years later by the same group [4] on the basis of a single-crystal X-ray diffraction experiment (Figure 1). Epi-hippodamine (**2**) is its unnatural isomer with an axial C-5 methyl group. Both hippodamine (**1**) [5–7] and epi-hippodamine (**2**) [8] have been synthesized previously, and we reported syntheses of these two compounds using a two-directional synthesis / tandem, reaction approach in 2005 [9]. Scheme 1 details the key aspects to our earlier work [10]. Herein, we report two refinements to our earlier work which have allowed even more concise routes to azaphenalene alkaloids **1** and **2**.

## Results and Discussion

**Scheme 2:**
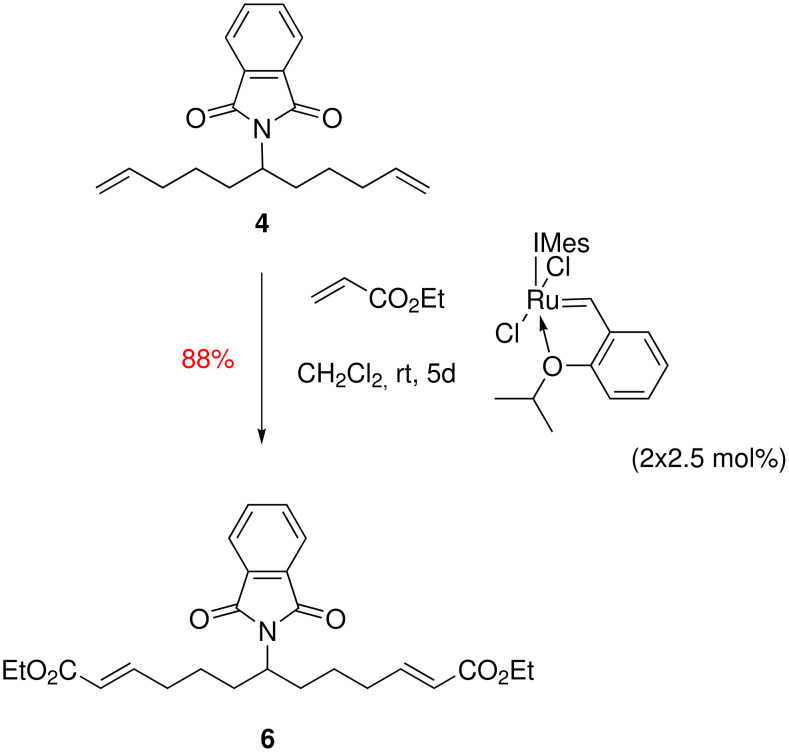
Improved synthesis of tandem reaction precursor **6**.

When we decided to take a second look at the syntheses of hippodamine and epi-hippodamine, we decided to focus on the synthesis of the key common intermediate **7** and try to realise an improvement over our earlier work. This paper discloses two such improvements. The first of these is the conversion of dialkene **4** into the diacrylate derivative **6** . Originally this was achieved by oxidative cleavage of the two alkene moieties of **4** to form the rather sensitive dialdehyde **5** . Whilst we were able to purify compound **5**, this resulted in a significant loss of material through degradation of the dialdehyde on the purification media, be it silica gel or neutral alumina. Thus, we found that use of the crude dialdehyde in the subsequent Horner-Wadsworth-Emmons reaction was preferable, and gave a good 75% yield of the doubly homologated compound **6** after purification by column chromatography. Whilst this process did allow us to produce multigram quantities of **6**, this sequence of reactions had the drawbacks that the olefination reaction needed to be carried out immediately after isolation of the dialdehyde **5** (this was found to decompose upon storage, even at low temperature), and also the oxidative cleavage reaction produced large amounts of toxic osmium waste. During our recent synthesis of histrionicotoxin [11], we found that two-directional homologation of a symmetrical dialkene similar to **4** using cross-metathesis with acrylonitrile was possible using the Hoveyda modification of Grubbs second generation catalyst [12] (Scheme 2). Thus, it seemed a logical extension of this thinking to see if we could carry out a direct double homologation of dialkene **4** with ethyl acrylate as the cross-metathesis coupling partner. In fact, due to the non-co-ordinating nature of ethyl acrylate (in comparison to acrylonitrile) and the inert phthalimide group, this reaction proved to be an outstanding success, delivering diester **6** in 88% yield over one step after a five day reaction in dichloromethane. This step therefore reduces the overall number of steps for the synthesis of hippodamine to eight, and increases the overall yield from 8 to 10%. Similarly it reduces our synthesis of epi-hippodamine to eleven steps and increases the yield from 13 to 16% overall. The two-directional cross-metathesis reaction is shown in Scheme 2.

**Scheme 3:**
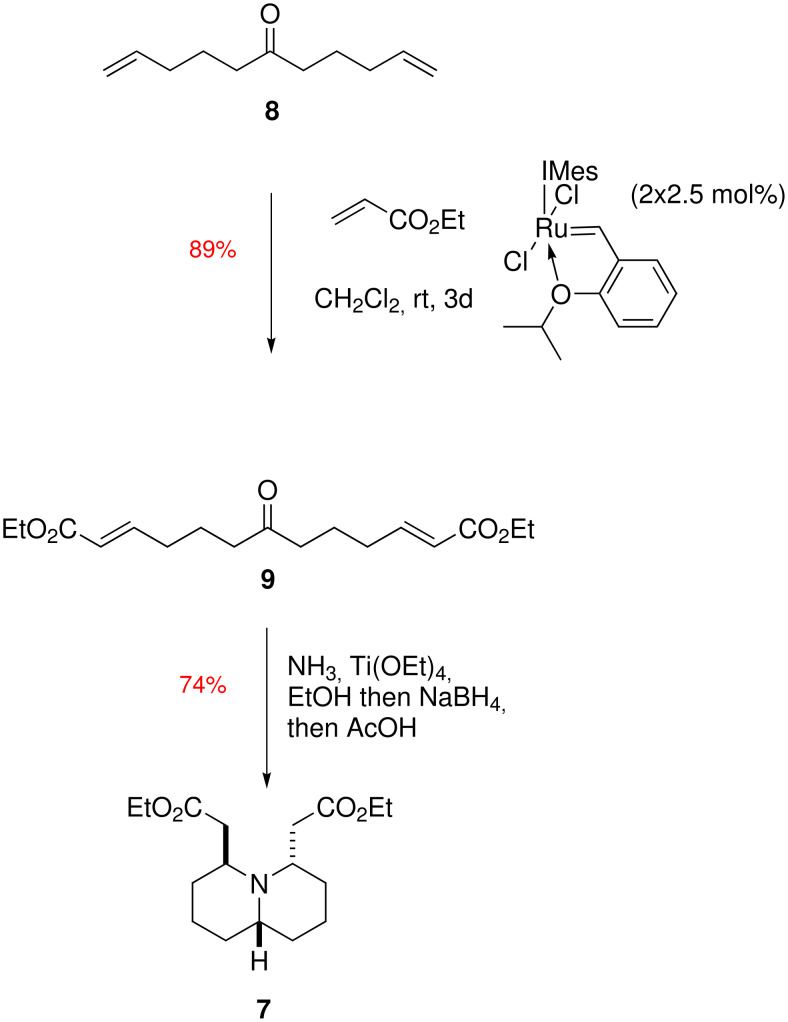
Tandem reductive amination / double intramolecular Michael addition.

Having refined our synthesis of diester **7** by providing a shortened synthesis of its precursor, we decided to see if we could attain a synthesis of this common intermediate for the synthesis of both hippodamine and epi-hippodamine without the use of the phthalimide protecting group. This would render the entire synthesis of hippodamine free of protecting group chemistry – a distinct driving force for a compound which may find use as an agrochemical. Thus we postulated whether we would be able to transform keto-diester **9** into quinolizidine **7** by a tandem reductive amination / double intramolecular Michael addition. Our results are shown in Scheme 3.

Thus ketone **8** was formed by reaction of the commercially available hex-5-enylnitrile with 4-pentenylmagnesium bromide in 70% yield [13]. Double cross-metathesis was found to proceed smoothly in 89% yield using the Hoveyda-Grubbs second generation catalyst in dichloromethane at room temperature for 3 days, giving ketodiester **9** [14]. We tried a range of reductive amination conditions for the formation of quinolizidine **7**. The ammonia equivalents tried were ammonium acetate, ammonium chloride and ammonium formate, along with sodium borohydride, sodium cyanoborohydride and Hantzsch ester in either ethanol or ethanol / acetic acid solvent systems. A summary of conditions tried is shown in Table 1. The ketodiester **9** was dissolved in ethanol and the ammonia source and desiccant were added and allowed to stir overnight to form the iminium species, before the hydride source was added and the reaction allowed to proceed for a further 24 h. The hydride source was quenched with acetone before excess glacial acetic acid was added and the reaction mixture was heated for the given time before quenching with brine. Entries 1–4 and entry 6 showed reduction of the ketone to the alcohol (by ^1^H NMR), and entry 5 using the Hantzsch ester showed no reaction at all.

**Table 1:** Summary of our efforts to effect quinolizidine formation in a one-pot reaction.

Entry	Amine Source	Desiccant	Hydride reagent	Temp (°C)	Time (h)	% Yield

1	NH_4_Cl and NEt_3_	-	NaBH_4_	75	48	-
2	NH_4_Cl and NEt_3_	-	Na(BH_3_CN)	80	48	-
3	NH_3_ in EtOH	4Å Sieves	Na(BH_3_CN)	rt	96	-
4	NH_4_OAc and NEt_3_	4Å Sieves	Na(BH_3_CN)	60	24	-
5	NH_4_OAc and NEt_3_	4Å Sieves	Hantzsch Ester	60	24	-
6	HCO_2_NH_4_ and NEt_3_	4Å Sieves	Na(BH_3_CN)	75	24	-
7	NH_3_ in EtOH	Ti(OEt)_4_	NaBH_4_	75	48	74

It was found that addition of ammonia in ethanol with titanium ethoxide [15] for 14 hours, followed by addition of sodium borohydride and stirring at room temperature for a further 8 hours, and finally the addition of acetone (to remove any remaining active hydride) and 30 equivalents of acetic acid followed by heating the reaction mixture at reflux for 48 hours gave a clean reaction as monitored by TLC to quinolizidine **7**, giving a 74% yield after purification by column chromatography over Brockmann Grade (III) neutral alumina. See Supporting Information File 1 for full experimental data. The tandem reductive amination / double intramolecular Michael addition generates 6 new bonds, 2 stereogenic centres and two rings, giving a single diastereomer.

In conclusion, we have increased the yield of our original hippodamine synthesis and reduced the number of steps required using a two-directional cross-metathesis of dialkene **4** with ethyl acrylate. We have also reported a new tandem reductive amination / double intramolecular Michael addition, which forms directly the quinolizidine core of hippodamine in a single step from a symmetrical keto-diester linear precursor. This new tandem reaction also reduces the number of steps for the synthesis of hippodamine to seven, and also removes any protecting group chemistry from the synthetic sequence and reduces waste whilst equalling the yield of the previous approach.

## Supporting Information

File 1 Experimental. Experimental procedures for compounds **4**,**6**,**7**,**9**.
