# Supplementary material for: Combining two-directional synthesis and tandem reactions, part 11: second generation syntheses of (±)-hippodamine and (±)-epi-hippodamine
Source: Beilstein J Org Chem. 2008 Jan 17;4:4. doi: 10.1186/1860-5397-4-4 (PMC2238745; doi:10.1186/1860-5397-4-4)
Supplement: File 1 — Experimental. Experimental procedures for compounds 4,6,7,9. [file Beilstein_J_Org_Chem-04-04-s001.doc]

**Combining two-directional synthesis and tandem reactions, part 11: second generation syntheses of (±)-hippodamine and (±)-epi-hippodamine**

*Annabella F. Newton,^a^ Martin Rejzek,^b^ Marie-Lyne Alcaraz^c^ and Robert A. Stockman*^a^*

**Experimental Section**

**General Procedures.** All chemicals were purchased as reagent grade and used without further purification. Tetrahydrofuran was freshly distilled from sodium diphenyl ketyl just prior to use. Dichloromethane was freshly distilled over CaH_2_. Other solvents for experiments were purified by the usual methods or purchased from a commercial source. If not stated otherwise TLC were performed on precoated silica plates containing a fluorescence indicator. Silica gel (63 – 200 μm) was used for analytical and preparative TLC (PLC) and for column chromatography, unless otherwise noted. Compounds were visualised under UV (254 nm), by heating after dipping in a solution of alkaline KMnO_4_ or by exposing to iodine vapours. NMR spectra were recorded on 300 or 400 MHz instruments. Chemical shifts are reported in ppm with respect to TMS. Coupling constants are reported in Hz. IR spectra of neat (unless otherwise noted) compounds were expressed as wavenumbers (cm^-1^). Water- and air-sensitive reactions were routinely carried out under argon atmosphere.

**2-(Undeca-1,10-dien-6-yl)isoindoline-1,3-dione** **(4).** A 2-necked 1000 mL flask equipped with addition funnel and magnetic stirrer was charged with triphenyl phosphine (42.9 g, 163.4 mmol), phthalimide (24.0 g, 163.4 mmol), anhydrous THF (700 mL) and undeca-1,10-dien-6-ol (**3**) (25 g, 148.6 mmol). The mixture was cooled to –20 °C under stirring and argon atmosphere. Diisopropyl azodicarboxylate (33.0 g, 32.4 mL, 163.4 mmol) in anhydrous THF (100 mL) was added dropwise over 1 h. When the addition was finished the mixture was stirred overnight at room temperature. The solvent was evaporated under reduced pressure and the oily residue was purified by three subsequent crystallisations from first EtOAc, then Et_2_O and finally petroleum ether-Et_2_O (1:1) in order to remove triphenylphosphine oxide. The resulting oily mixture was purified by column chromatography on silica gel (stepwise gradient of petroleum ether-Et_2_O 20:3 to pure Et_2_O) to afford **4** (42.41 g, 96.0%) as a yellow oil: *R_f_* 0.54 (petroleum ether-EtOAc 10:1); IR (cm^-1^) 3076, 3040, 2927, 2860, 1772, 1708, 1641, 1614, 1468, 1459, 1441, 1396, 1372, 1334; ^1^H NMR (400 MHz, CDCl_3_) 7.85-7.82 (2H, m), 7.73-7.70 (2H, m), 5.74 (2H, ddt, *J* = 16.8, 10.0 and 6.8), 5.01-4.90 (4H, m), 4.26-4.18 (1H, m), 2.16-1.99 (6H, m), 1.78-1.69 (2H, m), 1.51-1.23 (4H, m); ^13^C NMR (75 MHz, CDCl_3_) 169.0 (2C, s), 138.6 (2C, d), 134.1 (2C, d), 132.1 (2C, s), 123.3 (2C, d), 115.0 (2C, t), 52.0 (d), 33.4 (2C, t), 31.9 (2C, t), 26.0 (2C, t); MS (EI) *m/z* 297 (43, M^+^), 256 (47), 242 (25), 228 (29), 159 (40), 147 (100), 129 (63), 81 (65), 41 (76); HRMS calcd for C_19_H_27_N_2_O_2_ (M+NH_4_) 315.2073, found 315.2072.

**(2*E*,11*E*)-Diethyl 7-(1,3-dioxoisoindolin-2-yl)trideca-2,11-dienedioate (6)**

To a stirring solution of 2-(undeca-1,10-dien-6-yl)isoindoline-1,3-dione (**4**) (0.168 mmol, 50 mg, 1 eq) in dichloromethane was added ethyl acrylate (1.02 mmol, 0.11 mL, 6 eq) followed by Hoveyda-Grubbs 2^nd^ generation catalyst[1] (0.0042 mmol, 3 mg, 2.5 mol%) as a solid. The solution was stirred for at room temperature 96 h, monitoring by TLC, after which time another portion of catalyst (0.0042 mmol, 3 mg, 2.5 mol%) was added. The reaction mixture was stirred at room temperature for a further 24 h, at which point it was concentrated and subjected to purification by column chromatography (over SiO_2_ eluting with 6:1 H / EA) yielding the product **6** (0.149 mmol, 66 mg, 89%) as a clear yellow oil: R_f_ 0.6 (SiO_2_, 2:1 H / EA); MS (EI/CI): m/z [m+Na]+ 464.2 (100%) HRMS calculated for C_25_H_31_NNaO_6_ (M+Na) 464.2044, found 464.2069; δ_H_ (270 MHz; CDCl_3_) 7.79 - 7.84 (2H, m) 7.69 - 7.74 (2H, m) 6.84 (2H, dd, *J* 15.70 and 7.02) 5.79 (2H, dt, *J* 15.70 and 1.45) 4.14 (4H, q, *J* 7.16) 2.03 - 2.25 (5H, m) 1.61 - 1.79 (4H, m) 1.33 - 1.46 (4H, m) 1.19 - 1.30 (6H, m); δ_C_ (270 MHz, CDCl_3_) 166.7, 148.4, 134.1, 131.7, 123.4, 121.8, 60.3, 51.5, 32.0, 31.8, 25.1, 14.3; IR (thin film, ν/cm^-1^) 1706.

**(2*E*,11*E*)-Diethyl 7-oxotrideca-2,11-dienedioate (9).**

To a stirring solution of undeca-1,10-dien-6-one[2] (**8**) (0.60 mmol, 0.10 g, 1 eq) in dichloromethane (5 mL) under argon, was added ethyl acrylate (3.61 mmol, 0.36g, 6 eq), followed by Hoveyda-Grubbs 2^nd^ generation catalyst[1] (0.015 mmol, 9 mg, 2.5 mol%) as a solid. The solution was stirred for 24 h, at which time another portion of catalyst (0.015 mmol, 9 mg, 2.5 mol%) was added. The solution stirred for a further 48 h, concentrated, and purification by column chromatography (over SiO_2_ eluting with 6:1 PE/EA) gave **9** as a clear oil (0.54 mmol, 168 mg, 90%) MS (EI/CI): *m/z* [M+NH_4_]+ 328.3 (100%) HRMS calculated for C_17_H_30_NO_5_ (M+NH_4_) 328.2118, found 328.2117. δ_H_ (400 MHz; CDCl_3_) 6.91 (2H, dt, *J=* 15.7 and 7.1), 5.82 (2H, d, *J=* 15.7), 4.19 (4H, q, *J=* 7.3), 2.42 (4H, t, *J=* 7.4), 2.21 (4H, m),1.75 (4H, m), 1.29 (3H, t, *J=* 7.3); δ_C_ (100 MHz, CDCl_3_) 209.5, 166.5, 147.9, 122.1, 60.3, 41.8, 31.4, 21.9, 14.3; IR (thin film, ν/cm^-1^) C=O 1712, 1654.

**(4*S**, 6*S**)-(6-Ethoxycarbonylmethyloctahydroquinolizin-4-yl)acetic acid ethyl ester (7)**

(2*E*,11*E*)-Diethyl 7-oxotrideca-2,11-dienedioate **9** (0.16 mmol, 50 mg, 1 eq) was dissolved in ethanolic ammonia (2.0 M solution, 0.81 mmol, 0.41 mL, 5 eq) and titanium(IV) ethoxide (0.32 mmol, 0.07 mL, 2.5 eq) was added with stirring. The solution was stirred at room temperature for 14 h, after which time sodium borohydride (0.24 mmol, 9 mg, 1.5 eq) was added. The reaction was allowed to proceed for a further 8 h, at which time the excess hydride reagent was quenched by the addition of an aliquot of acetone (0.05 mL) and glacial acetic acid (4.8 mmol, 0.28 mL, 30 eq) was added. The reaction mixture was stirred at 75 °C for 48 h, after which time the reaction was quenched with brine. The solid residues were removed by filtration, washed with ethyl acetate (25 mL) and the aqueous layer extracted with ethyl acetate (4 x 25 mL) The combined organics were washed with brine, dried over Na_2_SO_3_ and the concentrated. The residue was redissolved in benzene and K_2_SO_3_ was added. The mixture was stirred for 18 h, at which time the solids were filtered off and the solvent removed. Purification by column chromatography (over activity III neutral Al_2_O_3_ eluting with 30:1 hexanes / ethyl acetate) yielded **7** (37 mg, 74%) as a clear yellow oil: R_f_ 0.79 (Al_2_O_3_, 5:1 hexanes / ethyl acetate) m/z [m+H]+ 312.2 (100%) HRMS calculated for C_17_H_30_NO_4_ (M+H) 312.2169, found 312.2179; ^1^H NMR (400 MHz, benzene-D6) 4.04-3.93 (4H, m), 3.83-3.80 (1H, m), 2.81 (1H, dd, *J =* 14.2 and 4.4), 2.78-2.72 (1H, m), 2.65 (1H, dd, *J =* 14.6 and 3.2), 2.41 (1H, dd, *J =* 14.6 and 10.4), 2.29 (1H, dd, *J =* 14.2 and 6.8), 1.98-1.92 (1H, m), 1.72-1.66 (3H, m), 1.47-1.36 (2H, m), 1.34-1.29 (4H, m), 1.24-1.04 (3H, m), 1.02-0.98 (6H, m); ^13^C NMR (75 MHz, benzene-D6) 171.9 (s), 170.9 (s), 59.0 (t), 58.9 (t), 53.9 (d), 52.9 (d), 50.0 (d), 38.5 (t), 33.7 (t), 33.6 (t), 32.0 (t), 28.6 (t), 26.3 (t), 22.9 (t), 17.7 (t), 12.9 (2C, q); IR (thin film, ν/cm^-1^) 2931, 2360, 1726.

**References**

Garber SB, Kingsbury JS, Gray BL, Hoveyda AH: *J. Am. Chem. Soc.* 2000, **122:** 8168-8179.

Fürstner A, Thiel OR, Kindler N, Bartowska B: *J. Org. Chem.* 2000, **65:** 7990-7995.
